# Arthroscopic rotator cuff repair in patients over 65 years of age: successful functional outcomes and a high tendon integrity rate can be obtained after surgery

**DOI:** 10.1016/j.jseint.2023.11.010

**Published:** 2023-12-08

**Authors:** Olimpio Galasso, Michele Mercurio, Giorgio Gasparini, Orlando Cosentino, Alessandro Massarini, Nicola Orlando, Roberto Castricini

**Affiliations:** a Department of Orthopaedic and Trauma Surgery, “Magna Græcia” University, Mater Domini” University Hospital, Catanzaro, Italy; b Division of Orthopaedic and Trauma Surgery, “Villa Maria Cecilia Hospital”, Cotignola, Italy

**Keywords:** Rotator cuff tears, Tendon integrity, Repair integrity, Arthroscopy, Range of motion, Constant-Murley Score, American Shoulder and Elbow Surgeons (ASES) score, SF-12 questionnaire

## Abstract

**Background:**

Although interest in studies evaluating the outcomes of rotator cuff repair is steadily increasing, the results and tendon integrity after arthroscopic rotator cuff repair in elderly patients have only been minimally investigated. The aim of this study was to evaluate clinical outcomes and repair integrity in patients over 65 years of age who underwent arthroscopic repair of full-thickness rotator cuff tears.

**Methods:**

A retrospective study was conducted with the following inclusion criteria: (1) elective shoulder arthroscopy for rotator cuff repair for full-thickness posterosuperior tears; (2) age over 65 years at surgery; and (3) participation in 24 months of follow-up. Preoperatively, the range of motion (ROM) and the Constant-Murley Score (CMS) and at follow-up, the ROM, the 12-Item Short Form Survey, the American Shoulder and Elbow Surgeons, and the CMS were evaluated; an ultrasonographic assessment of tendon integrity was performed according to the adapted Sugaya classification.

**Results:**

The final sample consisted of 110 patients with an average age of 69.2 ± 3.5 years. The mean duration of nonoperative management before surgery was 2.6 ± 0.8 months. The mean period of preoperative physical therapy was 0.6 ± 0.9 months. ROM and CMS showed statistically significant improvement (all *P* < .001) after a mean follow-up time of 54.5 ± 22.3 months. The ultrasonographic assessment showed tendon integrity (types I and II) in 75% of cases; 21% were type III repair, and rotator cuff retear (types IV and V) was recorded in 4% of cases. All scores directly correlated with the integrity of the tendon. In the multivariate analysis, higher postoperative CMS was associated with male sex (*P* < .001, β = −6.085) and lower age (*P* = .004, β = −0.533). Higher postoperative American Shoulder and Elbow Surgeons were associated with lower age (*P* = .020, β = −0.414). Higher postoperative 12-Item Short Form Survey physical component score and mental component score were associated with lower age (*P* = .013, β = −0.550 and *P* < .001, β = −0.520, respectively) and shorter preoperative physical therapy period (*P* = .013, β = −2.075 and *P* = .006, β = −1.093, respectively).

**Conclusion:**

A significant ROM and CMS recovery and a rotator cuff integrity rate of 75% can be expected in patients over 65 years of age who undergo arthroscopic repair for full-thickness rotator cuff tears. Better functional, physical, and mental health outcomes correlate with rotator cuff integrity and are predicted by male sex and a shorter period of preoperative physical therapy.

Rotator cuff tears are a common cause of shoulder pain and disability in the elderly population and affect approximately 20% of patients over 65 years of age.^27^

Management of rotator cuff tears consists of conservative treatment since symptoms may resolve or become tolerable over time and some patients have low functional demands and an interest in pursuing nonsurgical management.^7^ Therefore, most patients are initially prescribed physical therapy, nonsteroidal anti-inflammatory drugs, and/or analgesics prior to referral for surgery.^12^ Although most cases respond to nonoperative management, surgery may become necessary in cases in which conservative treatment has failed. However, performing surgical procedures on elderly patients may be a challenge. In 1995, Hattrup and Scottsdale^16^ suggested that patients 65 years of age or older present a significant chance of having larger tears, and this may increase the difficulty of repair or result in a need for additional surgical procedures.^9^ The bone quality of elderly patients is lower and may complicate suture anchor fixation.^22^ Furthermore, elderly patients frequently present comorbidities that may diminish the healing response, compromising outcomes further.^27^ The literature suggests that there is a correlation between the integrity rate of the repair and the quality of functional outcomes and that the integrity rate decreases with age^17^; some authors have proposed that, due to muscle atrophy, tear size, and poor healing rates in elderly patients, rotator cuff tears in such patients could be treated with acromioplasty and biceps tenotomy alone.^17^ Although interest in studies evaluating the outcomes of rotator cuff repair is steadily increasing,^6^ the results and tendon integrity after arthroscopic rotator cuff repair in elderly patients have only been minimally investigated.^17^

The aim of this study was to evaluate clinical outcomes and repair integrity in patients over 65 years of age who underwent arthroscopic repair of full-thickness rotator cuff tears.

We hypothesize that patients over 65 years of age have successful outcomes in terms of range of motion (ROM) and functional recovery after arthroscopic repair for full-thickness rotator cuff tears and that better outcomes correlate with rotator cuff integrity.

## Material and methods

A retrospective study was conducted with prospective data collection on 120 patients who underwent shoulder arthroscopy for rotator cuff tear repair between July 2009 and February 2020. The study protocol was approved by the local ethics committee, and the research was conducted in compliance with the principles set forth in the Declaration of Helsinki. Informed consent was obtained from all participants included in the study. The inclusion criteria were (1) primary and elective shoulder arthroscopy for rotator cuff repair for full-thickness posterosuperior tears diagnosed using a combination of injury history, physical examination, and magnetic resonance imaging^26^; (2) age over 65 years at surgery; and (3) participation in follow-up for a minimum of 24 months. The exclusion criteria were (1) irreparable full-thickness posterosuperior tears with fatty infiltration greater than stage III and tendon retraction greater than Patte grade 2^24^; (2) revision rotator cuff repair surgery or prior shoulder surgery; (3) fracture or dislocation of the shoulder; (4) glenohumeral osteoarthritis; (5) neurological disorder of the upper extremities; (6) significant cognitive impairment; and (7) failure to understand or complete the questionnaires. Ten patients did not participate in the follow-up. Therefore, 110 of 120 patients were enrolled and evaluated.

The data gathered included the patient’s age, sex, dominant arm, comorbidities, preoperative and postoperative physical therapy, and any other preoperative or postoperative treatment.

### Surgical technique

All surgical procedures were performed by a single surgeon (R.C.) with extensive experience in shoulder arthroscopy. An interscalene block was performed for all procedures.

All patients were placed in the standard lateral decubitus position with the arm under longitudinal traction for routine arthroscopy, one suture anchor with two strands (Fastin RC; DePuy Mitek, Raynham, MA, USA or TwinfixTi; Smith & Nephew, Andover, MA, USA) was implanted at the bone-cartilage junction, and a modified lasso-loop stitch technique was used in all cases. Acromioplasty was used as an additional surgical procedure when the critical shoulder angle was higher than 35°. The long head of the biceps was evaluated and, in cases of instability or lesions, was treated with tenotomy or tenodesis in very thin patients to avoid Popeye deformity.

Postoperatively, patients were given intravenous acetaminophen, nonsteroidal anti-inflammatory drugs, and oral opioids for pain relief.^2^ The arm was immobilized for 3 weeks in a shoulder abduction brace.^8^ Active and passive ROM exercises of the shoulder were initiated after shoulder brace removal.^23^ Light strengthening exercises were delayed until 8 weeks. Demanding activities and sports were authorized 6 months postoperatively.

### Functional and rotator cuff integrity assessment

Preoperatively, each patient was evaluated using the ROM assessment and the Constant-Murley Score (CMS).^4^ At minimum 24-month follow-up, each patient was evaluated using the ROM assessment, the 12-Item Short Form Survey (SF-12) questionnaire^13^ for measurement of health status, the American Shoulder and Elbow Surgeons (ASES) score,^28^ and the CMS. The CMS was normalized for sex and age using the following formula: normalized CMS = (raw CMS/normal CMS) x 100.^18^ All patients were evaluated for intraoperative and postoperative complications. Preoperative and postoperative patient’s assessment was performed by trained physicians who were unaware of the diagnosis and treatment.

At minimum 24-month follow-up evaluation, an ultrasonographic assessment of tendon healing was performed in all patients. Two musculoskeletal radiologists who were unaware of the patient’s clinical characteristics performed the assessment using a 12 MHz transducer (Toshiba Aplio XG; Toshiba Medical Systems Ltd., Crawley, United Kingdom). Rotator cuffs were classified according to the Sugaya classification^32^ adapted for ultrasonographic assessment.^1^ In detail, a healed cuff was considered to be type I and II repaired cuffs >2 mm in thickness with normal or partial hypoechogenicity echostructure; type III cuffs were considered repaired cuffs that had insufficient thickness (<2 mm) without discontinuity; and types IV and V cuffs were re-torn cuffs that showed minor or major full-thickness discontinuity.

### Statistical analysis

All data were collected, measured, and reported at an accuracy of 1 decimal place. The mean, standard deviation, and range are listed for continuous variables, and counts are listed for categorical variables. The distribution of the numeric samples was assessed using the Kolmogorov‒Smirnov normality test. Based on this preliminary analysis, parametric tests were adopted. Correlations were tested to investigate possible associations among the available data, and Pearson’s coefficient or Phi’s coefficient was adopted when appropriate. The correlation was considered to be strong (r > 0.5), medium (0.5< r < 0.3), or small (0.3 < r < 0.1). Univariate and multivariate linear regression was performed on the whole population to test possible outcome predictors. The explanatory and confounding preoperative and postoperative variables included in the analysis were sex (categorical), age (continuous), dominant limb (categorical), comorbidities (categorical), ROM (continuous), SF-12 (continuous), ASES (continuous), and follow-up (continuous). The postoperative functional score (a continuous variable) was treated as an outcome of the variables. Only explanatory and confounding variables that showed a trend toward an association (e.g., *P* < .10) with the outcome of interest in the univariate analysis were included in the multiple regression analysis.

Post hoc power was calculated by considering the sample size, the observed effect size, and an α-value of 0.05; a post hoc power greater than 80% was found, and it was considered appropriate. IBM SPSS Statistics software (version 26; IBM Corp., Armonk, NY, USA) and G∗Power (version 3.1.9.2; Heinrich Heine Universität, Düsseldorf, Germany) were used to construct the database and perform statistical analysis. A *P* value of less than 0.05 was considered significant.

## Results

The demographic characteristics of the included patients are summarized in Table I. The final sample consisted of 110 patients, 54 (49%) of whom were female, with an average age of 69.2 ± 3.5 years (range, 65-79 years) at surgery.

**Table I:** Baseline characteristics of included patients.

Patients (n = 110)	Mean ± SD (range) or n (%)
Gender	
Male	56 (51 %)
Female	54 (49 %)
Age at follow-up *(y)*	69.2 ± 3.5 (65-79)
Side	
Right	64 (58%)
Left	46 (42%)
Limb dominance	67 (61%)
Diabetes mellitus	12 (11%)
Hypercholesterolemia	27 (25%)
Benign prostatic hyperplasia	14 (13%)
Acute coronary syndrome	6 (5%)
Preop corticosteroid injections	11 (10 %)
Fluoroquinolone use	9 (8%)
Physical therapy preop (*mo*)	0.6 ± 0.9 (0-6)
Physical therapy postop (*mo*)	3.1 ± 2.0 (2-12)
Follow-up *(mo)*	54.5 ± 22.3 (24-151)

*SD*, standard deviation; *n*, number of cases; *preop*, preoperative; *postop*, postoperative.

The mean duration of nonoperative management before surgery was 2.6 ± 0.8 months (range, 2-6 months). The mean period of preoperative physical therapy was 0.6 ± 0.9 months (range, 0-6 months). Preoperative corticosteroid injection was performed in 10% of cases. During surgery, 20 patients received isolated arthroscopic rotator cuff repair. Acromioplasty was performed in 90.9% of cases, tenotomy in 74.7%, and tenodesis in 6.3%. No intraoperative complications occurred. The mean duration of postoperative physical therapy was 3.1 ± 2 months (range, 2-12 months).

After a mean follow-up time of 54.5 ± 22.3 months (range, 24-151 months), the mean SF-12 physical component score (PCS), SF-12 mental component score (MCS), and ASES scores were 56.6 ± 8.8 (range, 23 - 57), 60.8 ± 4.4 (range, 44 - 61), and 96.6 ± 6.5 (range, 72 – 100), respectively.

Table II shows the differences between flexion, abduction, and external rotation ROM and CMS values before surgery and at follow-up. All outcomes showed statistically significant improvement (all *P* < .001). Before surgery, the mean CMS was 58.8% that of sex- and age-matched healthy individuals, and all patients showed a CMS lower than the normative data. At the final follow-up visit, the mean CMS was 1.4% higher than that of sex- and age-matched healthy individuals, and 73.6% of patients showed a CMS equal to or higher than the normative data. The mean increase in the CMS was 33.4 ± 7.9 points (range, 2-50 points).

**Table II:** Differences in range of motion and Constant and Murley Score between preoperative and postoperative values.

Outcomes	Preoperative	Postoperative	*P* value	95% CI	SED
	Mean ± SD	Mean ± SD			
Flexion	160.5 ± 14.2	174.9 ± 12.7	**<.001**	**−16.98 to −11.84**	**1.3**
Abduction	145.4 ± 20.6	172 ± 17.1	**<.001**	**−30.59 to −22.59**	**2.02**
External rotation	21.1 ± 3.6	28 ± 4.7	**<.001**	**−7.92 to −5.90**	**0.51**
CMS pain	0.1 ± 0.8	13.9 ± 2.1	**<.001**	**−14.21 to −13.39**	**0.21**
CMS ADL	12.2 ± 1.4	19 ± 2.2	**<.001**	**−7.26 to −6.26**	**0.25**
CMS ROM	30.9 ± 4.1	38.6 ± 3.8	**<.001**	**−8.61 to −6.77**	**0.47**
CMS strength	1.6 ± 1.1	6.6 ± 1.9	**<.001**	**−5.38 to −4.64**	**0.19**
CMS total	44.7 ± 5.2	78.1 ± 7.7	**<.001**	**−34.87 to −31.90**	**0.75**

*CMS*, Constant Murley Score; *SD*, standard deviation; *CI*, confidence interval; *SED*, standard error of difference; *ADL*, activity daily living; *ROM*, range of motion.

Results with a *P* value of less than .05 are in bold.

At the follow-up, the ultrasonographic assessment showed tendon integrity (types I and II) in 75% of cases; 21% were type III repair, and rotator cuff retear (types IV and V) was recorded in 4% of cases. Cohen’s kappa coefficients for intraobserver and interobserver reliability of tendon healing were 0.88 and 0.85, respectively.

Additional file 1 shows the correlations between ultrasonographic classification of the repaired cuff and the SF-12 PCS, SF-12 MCS, ASES, and CMS assessment tools at follow-up. All scores directly correlated with each other and with the integrity of the tendon.

Table III shows the results of the univariate regression analysis.

**Table III:** Univariate regression analysis: factors affecting postoperative outcome scores and rotator cuff integrity.

β, regression coefficient	CMS		CMS recovery rate		ASES		SF-12 PCS		SF-12 MCS		Postoperative cuff integrity	
	β	*P* value	β	*P* value	β	*P* value	β	*P* value	β	*P* value	β	*P* value
Age	**−0.669**	**.001**	−0.272	.194	**−0.466**	**.007**	**−0.638**	**.005**	**−0.569**	**<.001**	−0.011	.475
Sex	**−7.309**	**<.001**	0.001	.999	**−3.659**	**.002**	**−4.569**	**.004**	**−1.955**	**.018**	−0.138	.189
Follow-up	0.063	.053	−0.055	.098	0.033	.231	0.052	.158	**0.038**	**.042**	0.001	.899
Acromial type	−0.080	.942	1.596	.153	0.703	.458	−0.076	.951	0.226	.722	0.109	.173
Dominant limb	1.620	.381	2.697	.073	2.156	.090	1.760	.291	−0.660	.441	0.072	.503
Preoperative CMS	**0.448**	**.001**	**−0.552**	**<.001**	**0.238**	**.048**	**0.426**	**.006**	**0.234**	**.003**	0.001	.466
Preop flexion ROM	**0.148**	**.004**	**−0.115**	**.026**	0.051	.247	0.103	.072	**1.624**	**<.001**	0.003	.385
Preop abduction ROM	**0.104**	**.003**	**−0.093**	**.010**	**0.061**	**.045**	**0.113**	**.003**	**0.058**	**.004**	0.001	.715
Preop external rotation ROM	0.346	.168	**−0.486**	**.019**	0.134	.441	0.352	.118	0.103	.377	0.001	.962
Preop physical therapy (mo)	−1.186	.222	0.693	.383	**−1.732**	**.009**	**−2.427**	**.005**	**−1.233**	**.005**	−0.026	.643
Postop physical therapy (mo)	−0.657	.153	−0.441	.241	**−0.685**	**.030**	−0.641	.121	−0.079	.709	−0.005	.854
Preop corticosteroid injection	−3.111	.301	0.081	.973	−0.614	.768	−2.695	.319	**−2.772**	**.045**	−0.273	.119
Arterial hypertension	−1.447	.433	0.355	.813	−0.671	.599	−1.189	.474	−0.993	.243	−0.030	.779
Hypercholesterolemia	1.733	.409	2.532	.138	−0.795	.584	−1.878	.319	−0.657	.499	0.054	.660
Benign prostatic hyperplasia	3.629	.179	0.704	.751	1.972	.292	2.838	.244	1.329	.288	−0.065	.679
Diabetes mellitus	−3.032	.295	−0.972	.681	−2.274	.169	−2.668	.306	−1.145	.393	−0.131	.439
Acute coronary syndrome	3.577	.368	0.584	.857	3.588	.190	4.422	.216	2.430	.186	0.141	.544
Fluoroquinolone use	−0.915	.782	−1.978	.462	1.880	.409	0.144	.961	−0.274	.858	0.084	.660

*CMS*, Constant and Murley Score; *ASES*, American Shoulder and Elbow Surgeons; *SF-12*, 12-Item Short Form Survey; *PCS*, physical component score; *MCS*, mental component score; *ROM*, range of motion.

Results with a *P* value of less than .05 are in bold.

In the multivariate analysis, higher postoperative CMS was associated with male sex (*P* < .001, β = −6.085) and lower age at surgery (*P* = .004, β = −0.533). This model accounted for 28.3% of the postoperative variance of the CMS.

Higher CMS recovery rate was associated with lower preoperative CMS (*P* = .002, β = −0.693). This model accounted for 11.7% of the variance in CMS recovery rate.

Higher postoperative ASES values were associated with lower age at surgery (*P* = .020, β = −0.414). This model accounted for 14% of the variance in postoperative ASES.

Higher postoperative SF-12 PCS values were associated with lower age at surgery (*P* = .013, β = −0.550) and shorter preoperative physical therapy period (*P* = .013, β = −2.075). This model accounted for 16.8% of the variance in postoperative SF-12 PCS.

Higher postoperative SF-12 MCS values were associated with lower age at surgery (*P* < .001, β = −0.520) and shorter preoperative physical therapy period (*P* = .006, β = −1.093). This model accounted for 29.5% of the variance in postoperative SF-12 MCS.

## Discussion

After a mean 4.5-year follow-up, a high rate of tendon integrity was recorded, and significant improvement in overall functional outcomes was reported. The postoperative CMS values of the patients in the study were similar to those of sex- and age-matched healthy individuals. Higher postoperative CMS, ASES, and SF-12 PCS and MCS were all associated with lower age at surgery. A shorter duration of preoperative physical therapy was associated with higher postoperative SF-12 scores, and male sex predicted higher postoperative CMS.

The study population evaluated in the present article had a mean age of 69.2 years. To the best of our knowledge, the population studied here represents one of the largest and longest series of rotator cuff repair in elderly patients. Overall, the successful results of this study confirm what Fossati et al^11^ found in their recent systematic review. Those authors reported a mean postoperative CMS of 71.5 points with a mean improvement of 31.2 points; this was greater than the minimal clinically important difference for CMS (10.4 points).^20^ A similar improvement was registered in ASES, and the ultrasonographic evaluation of rotator cuff integrity at follow-up showed a retear rate of 18.6%. The patients in the current study showed a mean statistically significant CMS recovery of 33.4 points. We found that higher postoperative CMS, ASES, and SF-12 PCS and MCS were all associated with lower age at surgery, suggesting that less favorable postoperative outcomes can be expected in older patients. There has been debate over whether age affects functional outcome after rotator cuff repair.^15^ Indeed, previous studies have reported favorable outcomes for both young^19^ and old^30^ patients. Our data were analyzed using multivariate analysis, and outcome scores all agree in predicting better results in relatively younger patients.

Considering the integrity of the repair at follow-up, we found an integrity rate of 75%. We used an ultrasonographic assessment ie, highly sensitive (83.7%) and specific (90.7%); as recently stated in a meta-analysis in which the two radiological methods were compared for their usefulness in diagnosing retear of a repaired rotator cuff tendon, it is as useful as magnetic resonance imaging.^14^ A similar integrity rate was reported by Jacquot et al,^17^ who found, as we did, better clinical outcomes in patients with intact tendons. Flurin et al^10^ reported that patients with re-torn cuffs had worse CMS and ASES scores, and Yoo et al^33^ also reported that SF-36 PCS as well as The University of California-Los Angeles shoulder scale and ASES scores were significantly higher in the healed group.

We failed to find an association between older age and retear rate, and we demonstrated that rotator cuff integrity remains high after surgery, even in elderly patients. Notably, chronological aging is not a precise marker of biological aging. The effect of advanced age on rotator cuff healing is still debated. Sixty-nine years of age is identified as a conventional cutoff value for successful healing after arthroscopic repair.^29^ Indeed, the retear rate, which increases minimally until 65 years of age, begins to rise substantially in patients over the age of 70 years^5^^,^^16^; nevertheless, controversy persists about whether structural repair integrity affects functional outcome. Park et al^29^ reported that although a relatively high retear rate can be expected in elderly patients, clinical outcomes still showed significant improvement after a mean follow-up of 3 years.

We also found that higher postoperative CMS was associated with male sex. This finding concurs with the data reported by Robinson et al;^31^ indeed, the authors of that study investigated outcomes after arthroscopic rotator cuff repair in patients over 70 years of age and found that male gender was significantly associated with higher CMS one year postoperatively. Similarly, Lam and Mok^21^ studied the outcome of open repair in patients over 65 years of age and reported that male sex and shorter duration of symptoms were related to better outcomes. Males also showed higher postoperative CMS after arthroscopic repair of partial articular supraspinatus tendon avulsion.^3^ Interestingly, we found that physical and mental health outcomes were associated with a shorter duration of preoperative physical therapy. This suggests that a long duration of symptoms and unsuccessful physical therapy prior to surgery may lead to patient frustration, compromising the postoperative outcome.^27^

### Limitations

The major limitations of the current study are its retrospective nature and the lack of a control group. The lack of preoperative administration of health status evaluation scales that were used at follow-up and the choice to not include a psychological evaluation other than the patient’s self-assessment represent additional weaknesses. Indeed, response bias due to self-reporting cannot be excluded, and mental well-being and functional recovery may have been influenced by preexisting mental health conditions.^25^ However, the prospective nature of the data collection methods, the use of the same surgical technique in all patients, the use of validated and standardized functional assessments, the use of ultrasonographic evaluation to assess the integrity of the repair after a minimum of 24 months, the statistical reliability produced by the regression analyses, and the fact that the sample size and follow-up are comparable to those in the largest and longest series currently available^11^ represent the considerable strengths of the present study. However, firm conclusions cannot be made and future prospective randomized controlled trials on this topic are needed.

## Conclusion

Our results showed that statistically and clinically significant ROM and CMS recovery and a rotator cuff integrity rate of 75% can be expected in patients over 65 years of age who undergo arthroscopic repair for full-thickness rotator cuff tears. Postoperative CMS values similar to those of sex- and age-matched healthy individuals can be achieved in elderly patients even if lower postoperative functional scores are recorded in older patients. Better functional, physical, and mental health outcomes correlate with rotator cuff integrity and are predicted by male sex and a shorter period of preoperative physical therapy.

## Disclaimers

Funding: No funding was disclosed by the authors.

Conflicts of interest: The authors, their immediate families, and any research foundation with which they are affiliated have not received any financial payments or other benefits from any commercial entity related to the subject of this article.
