# Supplementary material for: Arthroscopic rotator cuff repair in patients over 65 years of age: successful functional outcomes and a high tendon integrity rate can be obtained after surgery
Source: JSES Int. 2023 Dec 8;8(2):299–303. doi: 10.1016/j.jseint.2023.11.010 (PMC10920122; doi:10.1016/j.jseint.2023.11.010)
Supplement: Additional File 1 [file mmc1.docx]

**Additional file 1** Correlation analysis among postoperative outcome measures

|  | SF-12  PCS | SF-12  MCS | ASES | CMS |
| --- | --- | --- | --- | --- |
| SF-12 MCS | **0.459**  **(p<0.001)** |  |  |  |
| ASES | **0.801**  **(p<0.001)** | **0.346**  **(p<0.001)** |  |  |
| CMS | **0.521**  **(p<0.001)** | **0.272**  **(p<0.001)** | **0.613**  **(p<0.001)** |  |
| Postop cuff integrity | **0.134**  **(p<0.001)** | **0.106**  **(p<0.001)** | **0.172**  **(p<0.001)** | **0.149**  **(p<0.001)** |

SF-12 means 12-Item Short Form Survey; PCS physical component score; MCS mental component score; ASES, American Shoulder and Elbow Surgeons Shoulder Score; CMS, Constant and Murley score.
